# *Staphylococcus aureus*/*Staphylococcus epidermidis* from skin microbiota are balanced by Pomegranate peel extract: An eco-sustainable approach

**DOI:** 10.1371/journal.pone.0308211

**Published:** 2024-08-01

**Authors:** Sara D’Arcangelo, Paola Di Fermo, Firas Diban, Vincenzo Ferrone, Simonetta D’Ercole, Mara Di Giulio, Silvia Di Lodovico

**Affiliations:** 1 Department of Pharmacy, University “G. d’Annunzio” Chieti-Pescara, Chieti, Italy; 2 Department of Medical, Oral and Biotechnological Sciences, University “G. d’Annunzio” Chieti- Pescara, Chieti, Italy; University of Milan, ITALY

## Abstract

The imbalance in skin microbiota is characterized by an increased number of pathogens in respect to commensal microorganisms. Starting from a skin microbiota collection, the aim of this work was to evaluate the possible role of Pomegranate (*Punica granatum* L.) Peel Extract (PPE) in restoring the skin microbiota balance acting on *Staphylococcus* spp. PPE was extracted following green methodology by using n-butane and the Dimethyl Ether (DME) solvents and analyzed for phytochemical composition and antimicrobial activity. The PPE antimicrobial action was evaluated against Gram +, Gram − bacteria and yeast reference strains and the most effective extract was tested against the main skin microbiota isolated strains. PPE extracted with DME showed the best antimicrobial action with MICs ranging from 1 to 128 mg/mL; the main active compounds were Catechin, Quercetin, Vanillic acid and Gallic acid. The PPE in DME anti-adhesive effect was examined against *S*. *epidermidis* and *S*. *aureus* mono and dual-species biofilm formation by biomass quantification and CFU/mL determination. The extract toxicity was evaluated by using *Galleria mellonella* larvae *in vivo* model. The extract displayed a significant anti-adhesive activity with a remarkable species-specific action at 4 and 8 mg/mL against *S*. *epidermidis* and *S*. *aureus* mono and dual-species biofilms. PPE in DME could represent an eco-sustainable non-toxic strategy to affect the Staphylococcal skin colonization in a species-specific way. The innovation of this work is represented by the reuse of food waste to balance skin microbiota.

## Introduction

The skin represents the largest organ of the human body with a surface area of about 2 m^2^ that extends to 25 m^2^ taking into account all the skin appendages (about 5 million), including sweat ducts and hair follicles [1]; it represents a shield against the environmental pollutants, chemicals and microorganisms [2]. Healthy human skin is colonized by numerous microorganisms which constitute the skin microbiota that lives in equilibrium establishing a microbial eubiosis. Its composition depends on the body site, sex, age and skin pH value. Moreover, the body cleansing detergents, cosmetics, living environment and many other factors can affect skin microbiota [3, 4]. The main microorganisms of skin microbiota belong to *Staphylococcus*, *Corynebacterium*, *Streptococcus* and *Propionibacterium* genera. Among *Staphylococcus* spp., the main isolated species are coagulase-negative Staphylococci (CoNS), in particular *S*. *epidermidis* is the most reported strain. CoNS play a pivotal role in the skin microbial equilibrium maintenance, through the competition/inhibition of pathogens by regulating the local inflammatory response, promoting keratinocyte homeostasis, and maintaining the integrity of the epidermal layer [5].

In a healthy balanced skin microbiota, *S*. *hominis* produces antimicrobial peptides against *S*. *aureus* affecting the *agr* system, without interfering with the growth of other commensals like *S*. *epidermidis* [6]. *Staphylococcus lugdunensis* can produce a thiazoline-containing cyclic peptide antibiotic named lugdunin that inhibits the growth of pathogens like *S*. *aureus* [5, 7]. *Staphylococcus epidermidis* produces phenol-soluble gamma and delta modulins that show a selective anti-*S*. *aureus* activity [8] and bacteriocins with effect against methicillin-resistant *S*. *aureus* [9]. Finally, *S*. *epidermidis* metabolism produces butyric acid, a short-chain fatty acid that inhibits *in vitro* and *in vivo S*. *aureus* growth [10].

The perturbation of skin microbial equilibrium leads to dysbiosis which is a condition characterized by increase in pathogenic microbial load, decrease in commensals and change microbial diversity [11, 12].

Unbalanced skin microbiota is associated with skin diseases, such as acne, atopic dermatitis (AD), folliculitis, psoriasis or seborrheic dermatitis [13, 14]. In particular, the increase of *S*. *aureus* is related to the development of AD; in fact, as reported by Kim et al., *S*. *aureus* was frequently isolated from patients with moderate to severe AD (60–100% of individuals), compared to people with healthy skin [15]. Moreover, injured sites have a higher amount of *S*. *aureus* (70%) than sites with no lesions (39%) in AD patients [16].

Different approaches were proposed to preserve and control the microbial proliferation of the skin microbiota such as prebiotics, oats or other natural compounds [17, 18].

Pomegranate (*Punica granatum* L.) is a fruit widely used in the food industry, in particular to produce juices and extracts, and it is known for its many beneficial properties and its abundance in many countries [19]. Pomegranate Peel Extract (PPE) showed antibacterial activity against several bacterial strains, including *S*. *aureus* [20], *Escherichia coli* and *Pseudomonas aeruginosa* [21, 22]. This activity is attributed to the phenolic compounds belonging to the family of ellagitannin, such as ellagic acid, punicic acid, punicalagin and gallic acid, that cause bacterial cell damage leading to cell death [23].

Based on these considerations, in this work, it was evaluated the antimicrobial effects of PPE obtained by an innovative green extraction method. Starting from a skin microbiota samples collection, the antimicrobial and anti-adhesive effects of PPE were evaluated against skin microbiota strains and the possible species-specific activity was investigated. Finally, PPE toxicity test was performed using *Galleria mellonella* larvae *in vivo* model.

## Materials and methods

### Skin microbiota collection and microbial culture

This study was approved by the Inter Institutional Ethic Committee of University “G. d’Annunzio” Chieti-Pescara, Chieti, Italy (ID: richycnvw). Skin microbiota samples were collected between April and June 2022 from the skin of six healthy volunteers (three men and three women; age range was 20–32 years) and from 3 volunteers with skin alterations (atopic dermatitis, one man and two women; age range was 25–32 years) who provided a written informed consent for the study. Study participants did not receive any systemic or topical antibiotic therapies within four months before the sampling; volunteers declared they did not treat the affected area with creams or lotions in the four days prior to sampling and they only used neutral soap and water to clean the swabbing area. For healthy subjects, skin microorganisms were collected from back skin, according to Ogai et al. [24]. Similarly, for subjects with atopic dermatitis, microorganisms were collected from the local area of skin alteration.

For skin sampling, a 4.5 x 4.5 cm square on the chosen area was swabbed with sterile cotton swab previously soaked in sterile saline solution. Then, the cotton swab was gently swabbed ten times horizontally and ten times vertically on selected skin area, and the swab was immersed in 1 mL of sterile saline solution, vortexed for 2 minutes and 100 μL was spread on blood agar plates (Tryptic Soy agar-TSA, Oxoid, Milan, Italy, with 5% of sheep blood) and incubated at 37°C both in aerobic and anaerobic conditions for 3 and 5 days, respectively. Isolated colonies from aerobic and anaerobic cultures were identified by Vitek 2 system (BioMérieux, France) (S1 Table) and stored at -80°C. Among bacterial isolates, *S*. *aureus* DAS 68, *S*. *aureus* DLS 69, *S*. *aureus* SP 70, *S*. *epidermidis* DLS 29 and *S*. *epidermidis* DAS 31 were selected for the experiments (S2 Table).

### Pomegranate peel extraction and characterization

Pomegranate used in the study was harvested from a private farm in Abruzzo (Italy) and the peel was obtained following the manual removal of the aryls.

The extract was obtained from the internal and external pomegranate peel, through an eco-sustainable extraction method with low environmental impact according to Ciulla et al. [25]. The extraction of pomegranate peel was performed under two different conditions: Dimethyl Ether (DME) and n-butane. The setup for extraction with DME was as following: a condenser column temperature of 287 K, an evaporation chamber temperature of 318 K, and a liquefied DME flow rate of 9–10 mL/min. For extraction with n-butane, the setup was as following: a condensation column temperature of 283 K, an evaporation chamber temperature of 318 K, and a liquefied n-butane flow rate of 8–9 mL/min. The sample, consisting of approximately 3.5–3.7 g of peels, was placed into the extraction chamber using a cellulose thimble for physical support and filtration. Subsequently, a vacuum (approximately 5 × 10–3 MPa) was applied to remove air from the tubing. In each run, 40.0 g of eluent (n-butane or DME) was introduced, and the experiments were conducted for durations of 30 minutes, 1 h and 2.5 h, with fresh samples loaded each time. At the end of the extraction, the liquefied gas was extracted from the sample by vaporising it into a recovery chamber. The extract was stored under a nitrogen atmosphere at 275 K. A high-performance liquid chromatography (HPLC) system was used to chemically characterize the PPE. Separation was achieved using gradient elution of a mobile phase composed by a solution of water and 0.1% acetic acid (Line A) and a mixture of acetonitrile and 2-propanol (75–25%, v/v, Line B). Gradient elution starts with 98% of Line A, then in 25 minutes become 80%, and after 15 minutes become 35%, then remain constant for 10 minutes, after that become 5% in 2 minutes and remain constant for 5 minutes, finally, come back to the initial composition in 2 minutes and remain constant for 12 minutes for the re-equilibration step. Analytes were detected at their maximum wavelength; total run time was 70 minutes. Standard solutions of the investigated polyphenols were obtained by weighing an exact amount of each reference powder into an analytical balance and solubilizing them into a volumetric flask with methanol. Working standard solutions containing the analytes were obtained by diluting stock solution with a mixture of water and methanol (50–50%, v/v). The calibrators were obtained in the range 0.5–50 μg/mL by further dilution of the standard solutions. The samples to be analyzed were prepared using 50 μL of extract and diluting it to 500 μL with methanol. 20 μL was injected into the HPLC system. Calibration curve was obtained by linear regression analysis of concentrations (X-axis) to response (area, Y-axis) using Graph-Pad software. Before the microbiological tests, the characterized PPE was tyndallized at 70°C for 30 minutes three times and the sterility was checked by spreading on nutrient agar plates.

### PPE antimicrobial activity

To evaluate the most effective extraction method (DME or n-butane), PPE antimicrobial tests were performed against reference strains: *S*. *aureus* ATCC 6538, *Escherichia coli* ATCC 10536 and *Candida albicans* ATCC 10231. PPE antimicrobial action was evaluated by Minimum Inhibitory Concentration (MIC) and Minimum Bactericidal/Fungicidal Concentration (MBC/MFC) determination according to Clinical & Laboratory Standards Institute (CLSI) guidelines [26]. PPE in DME and PPE in n-butane were tested at concentrations ranged from 32 mg/mL to 0.06 mg/mL. *S*. *aureus* ATCC 6538, *E*. *coli* ATCC 10536, *C*. *albicans* ATCC 10231 were also used for experiments. All strains were cultivated in Tryptic Soy Broth (TSB, Oxoid, Milan, Italy) at 37°C for 24 h then the broth cultures were diluted 1:10 in TSB and refreshed for 2 h at 37°C in thermostatic bath in agitation (120 rpm). Then, bacterial cultures were adjusted to an Optical Density (OD600) of 0.12 (corresponding to ~ 10^7^ CFU/mL). *Candida albicans* was cultured in RPMI (Sigma Aldrich, Milan, Italy) plus 2% glucose and standardized at OD600 0.15 (corresponding to ~ 10^6^ CFU/mL).

The standardized bacterial cultures were diluted 1:100 in Mueller Hinton Broth (MHB, Oxoid, Milan, Italy), *C*. *albicans* was diluted 1:10 in RPMI with 2% of glucose and used for the experiments. 100 μL of each standardized broth culture plus 100 μL of each concentration of PPE in DME and PPE in n-butane were inoculated in a 96-wells microtiter plate and incubated for 24–48 h. As positive control, each strain was incubated with medium without PPE and as negative control, medium without PPE was included.

MBC/MFC were determined by sub-culturing 10 μL of suspensions from the non-turbid wells on Mueller Hinton agar (MHA, Oxoid, Milan, Italy) plates and on Sabouraud Dextrose Agar (SDA, Oxoid, Milan, Italy) plates, respectively.

Similarly, selected skin microbiota strains (*S*. *aureus* DAS 68, *S*. *aureus* DLS 69, *S*. *aureus* SP 70, *S*. *epidermidis* DLS 29, *S*. *epidermidis* DAS 31) were used for the MIC/MBC determinations of PPE in DME, the most performing extract. Moreover, these selected strains isolated from skin microbiota were characterized for their antibiotic susceptibility profiles (S2 Table).

### PPE anti-adhesive effect

The capability of PPE in DME to interfere with biofilm formation was evaluated against *S*. *aureus* DLS 69 and *S*. *epidermidis* DAS 31, mono and dual-species biofilm. PPE in DME displayed the best activity against the tested strains. The standardized bacterial cultures were inoculated with sub-MIC concentrations of PPE (1/2 MIC, 1/4 MIC, 1/8 MIC; MIC *S*. *aureus* DLS 69 = 16 mg/mL; MIC *S*. *epidermidis* DAS 31 = 32 mg/mL; for the dual species biofilm the MIC *S*. *aureus* DLS 69 + *S*. *epidermidis* DAS 31 = 16 mg/mL) in 96-wells flat bottomed microtiter plates and incubated for 3 h and 24 h at 37°C in aerobic condition. After incubation, the supernatant was removed and each well was gently rinsed with sterile water. Biofilms CFU/mL determination and biofilm biomass quantification were analyzed. Briefly, for the CFU/mL determination, the bottom of each well was scraped, diluted and spread on Mannitol Salt Agar (MSA, Mannitol Salt Agar, Oxoid, Milan, Italy). For the biofilm biomass quantification, the washed and dried biofilm was stained with 0.1% Crystal violet solution and the biomass was quantified by measuring absorbance at 570 nm with microplate ELISA reader (BioRad, Milan, Italy) [17].

### PPE toxicity test

The PPE in DME toxicity was evaluated by using *Galleria mellonella* larvae *in vivo* model according to Di Lodovico et al. [27]. A total of six groups of 10 *G*. *mellonella* larvae (weight between 0.2 and 0.3 g) were used in the toxicity test. Each larva was injected in the last left proleg with 10 μL of PPE or Phosphate Buffered Saline (PBS, Merk, KGaA, Darmstadt, Germany) or uninjected, by using 0.3 mL micro-fine needle insulin syringes. Four groups were treated with 1000 mg/kg of PPE, 500 mg/kg of PPE, 250 mg/kg of PPE, 125 mg/kg of PPE. Two control groups were included as control (one treated with PBS, the other untreated). The larvae were incubated at 37°C in Petri dishes in the dark for seven days. The survival rate was monitored each day. The larvae were considered dead when they turn black and were unresponsive to touch. During assays, larvae did not receive nutrition. The survival rate was calculated by using Kaplan-Meier curve [28].

### Statistical analysis

All the results were obtained from at least three independent experiments performed in duplicate and they are shown as means ± standard deviation. Values of *p* ≤ 0.05 were considered statistically significant. Differences among groups were evaluated with ANOVA test.

## Results

### Skin microbiota collection

**Fig 1:**
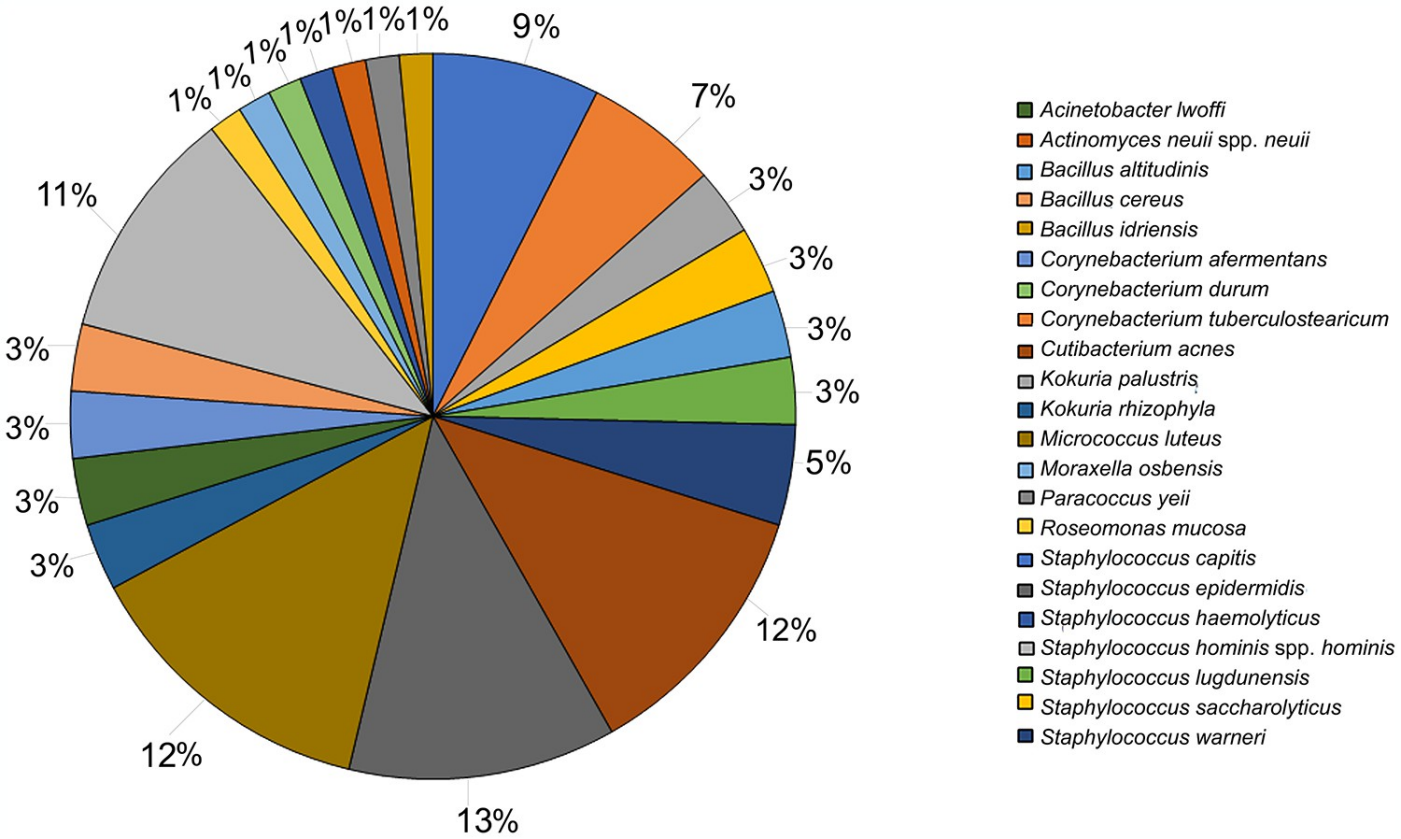
Skin microbiota collection. Percentage of skin microbiota species identified from back skin of healthy volunteers enrolled in the study.

From skin of healthy volunteers, 67 microorganisms were identified as reported in Fig 1 and S1 Table. The most abundant bacteria on the skin were *S*. *epidermidis* (13%), *Micrococcus luteus* (12%), *Cutibacterium acnes* (12%), *S*. *hominis* (11%).

**Fig 2:**
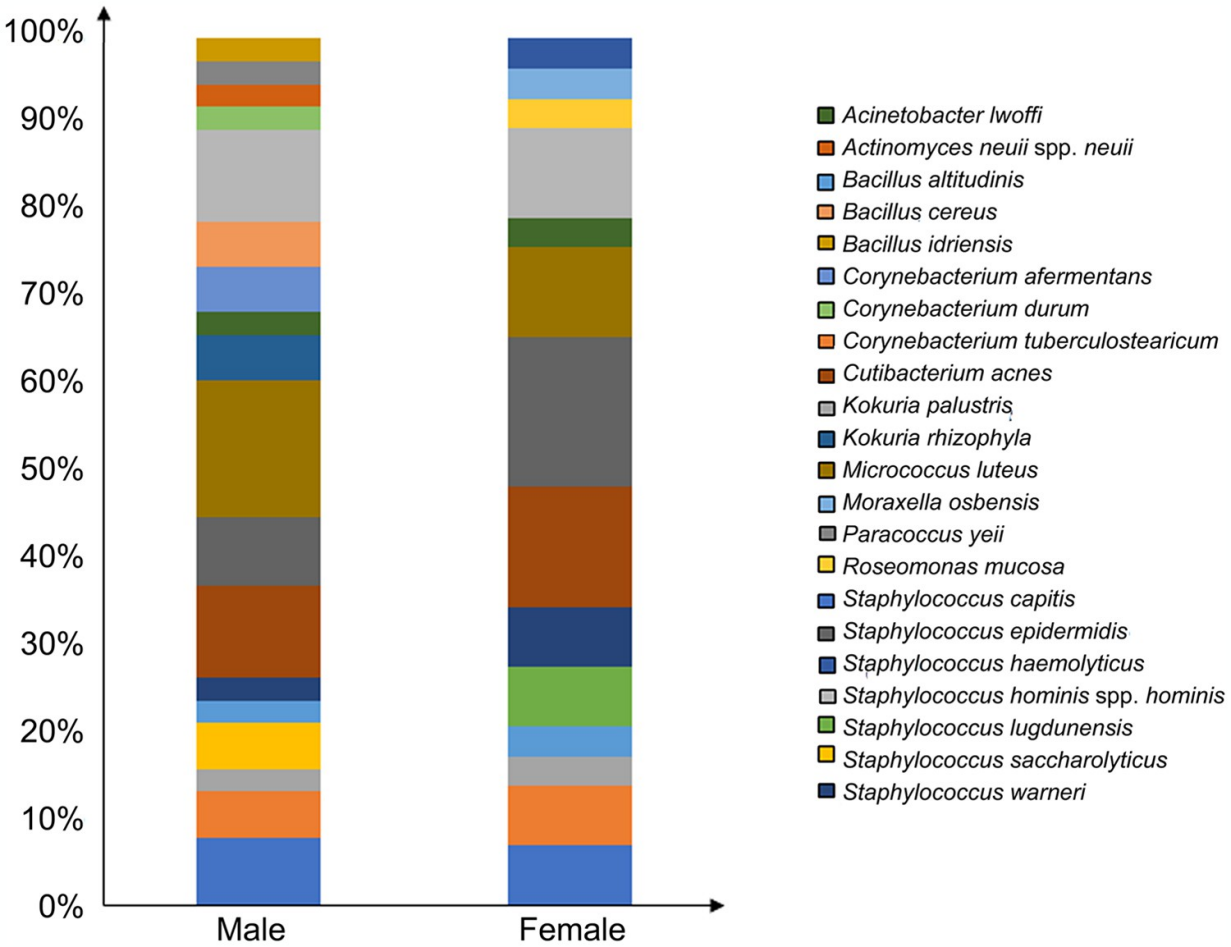
Bacterial abundance of skin microbiota in back skin of volunteers enrolled in the study: Male and female distribution.

The main relative abundance of bacterial species isolated from male and female volunteers, respectively, is shown in Fig 2. The main bacterial genus isolated from both sexes was *Staphylococcus* (35% and 51%, in male and female, respectively). The dominant taxonomic groups in male skin were: *M*. *luteus* (16%), *C*. *acnes* (11%), *S*. *hominis* (11%), *S*. *epidermidis* (8%), *S*. *capitis* (8%), while in female skin were: *S*. *epidermidis* (17%), *C*. *acnes* (14%), *S*. *hominis* (10%), *M*. *luteus* (10%), *S*. *capitis* (7%). *Staphylococcus saccharolyticus*, *Kokuria rhizophyla*, *Corynebacterium afermentans* and *Bacillus cereus* were only detected in men; *S*. *lugdunensis* was only detected in women.

*Corynebacterium durum* and *Paracoccus yeii* strains were only found in a volunteer from the Middle East region. Moreover, a volunteer that stated to have skin irritation problems in the past, harboured *Actinomyces neuii* spp. *neuii* and *B*. *idriensis*, which were not identified in the other analyzed subjects. This data shows a variability in skin microbiota among subjects who originated from different geographical regions or had skin changes. *Staphylococcus aureus* was the only strain isolated from AD volunteers.

### Pomegranate peel extraction and characterization

Table 1 displays the phytochemical characterization of PPE in DME after the tyndallization process; no differences in terms of antimicrobial activity were detected among tyndallized and non-tyndallized extract. As shown in Table 1, the most abundant components were represented by Catechin, Quercetin, Vanillic acid and Gallic acid.

**Table 1:** Phytochemicals composition of PPE in DME (concentrations are expressed in μg/mL).

Phytochemical compounds							
Gallic acid	Catechin	Vanillic acid	Chlorogenic acid	Caffeic acid	Rutin	Quercetin	Kaempferol
7.49	24.32	8.35	4.52	3.52	3.27	9.69	7.40

### PPE antimicrobial activity

The antimicrobial activity of both PPE extracted with DME and n-butane against the reference strains was evaluated. MIC and MBC/MFC of PPE in DME and n-butane against the reference strains, are listed in Table 2. MIC values ranged from 1 to 32 mg/mL. PPE in DME was the most active against bacteria with MIC values of 1 mg/mL for *S*. *aureus* ATCC 6538 and 2 mg/mL for *E*. *coli* ATCC 10536. PPE in n-butane was the most effective against *C*. *albicans* ATCC 10231. MBC/MFC values were all higher than 32 mg/mL. For these reasons, since bacterial species were the only microorganisms isolated from skin microbiota of volunteers enrolled in this study, the following tests were performed by using PPE in DME.

**Table 2:** Minimum Inhibitory Concentration (MIC) and Minimum Bactericidal/ Fungicidal Concentration (MBC/MFC) values of PPE in DME and n-butane against reference strains (concentrations are expressed in mg/mL).

	PPE in DME		PPE in n-butane	
Strains	MIC	MBC/MFC	MIC	MBC/MFC
***S*. *aureus* ATCC 6538**	1	>32	32	>32
***E*. *coli* ATCC 10536**	2	>32	16	>32
***C*. *albicans* ATCC 10231**	32	>32	4	>32

PPE: Pomegranate Peel Extract; DME: dimethyl ether.

Table 3 displays MIC and MBC of PPE in DME against the bacterial skin isolates. MIC values of clinical strains ranged from 16 mg/mL to 128 mg/mL; MBC values were all higher than 128 mg/mL. For *S*. *aureus* DLS 69 and *S*. *epidermidis* DAS 31 clinical strains the MIC values were 16 and 32 mg/mL, respectively. Among these strains, *S*. *aureus* DLS 69 and *S*. *epidermidis* DAS 31 were chosen to carry out the anti-adhesive activity test.

**Table 3:** Minimum Inhibitory Concentration (MIC) and Minimum Bactericidal Concentration (MBC) values (mg/mL) of PPE in DME of isolated skin bacteria.

PPE in DME		
Strains	MIC value	MBC value
***S*. *aureus* DAS 68**	128	>128
***S*. *aureus* DLS 69**	16	>128
***S*. *aureus* SP 70**	128	>128
***S*. *epidermidis* DLS 29**	128	>128
***S*. *epidermidis* DAS 31**	32	>128

PPE: Pomegranate Peel Extract; DME: dimethyl ether.

### PPE anti-adhesive activity

**Fig 3:**
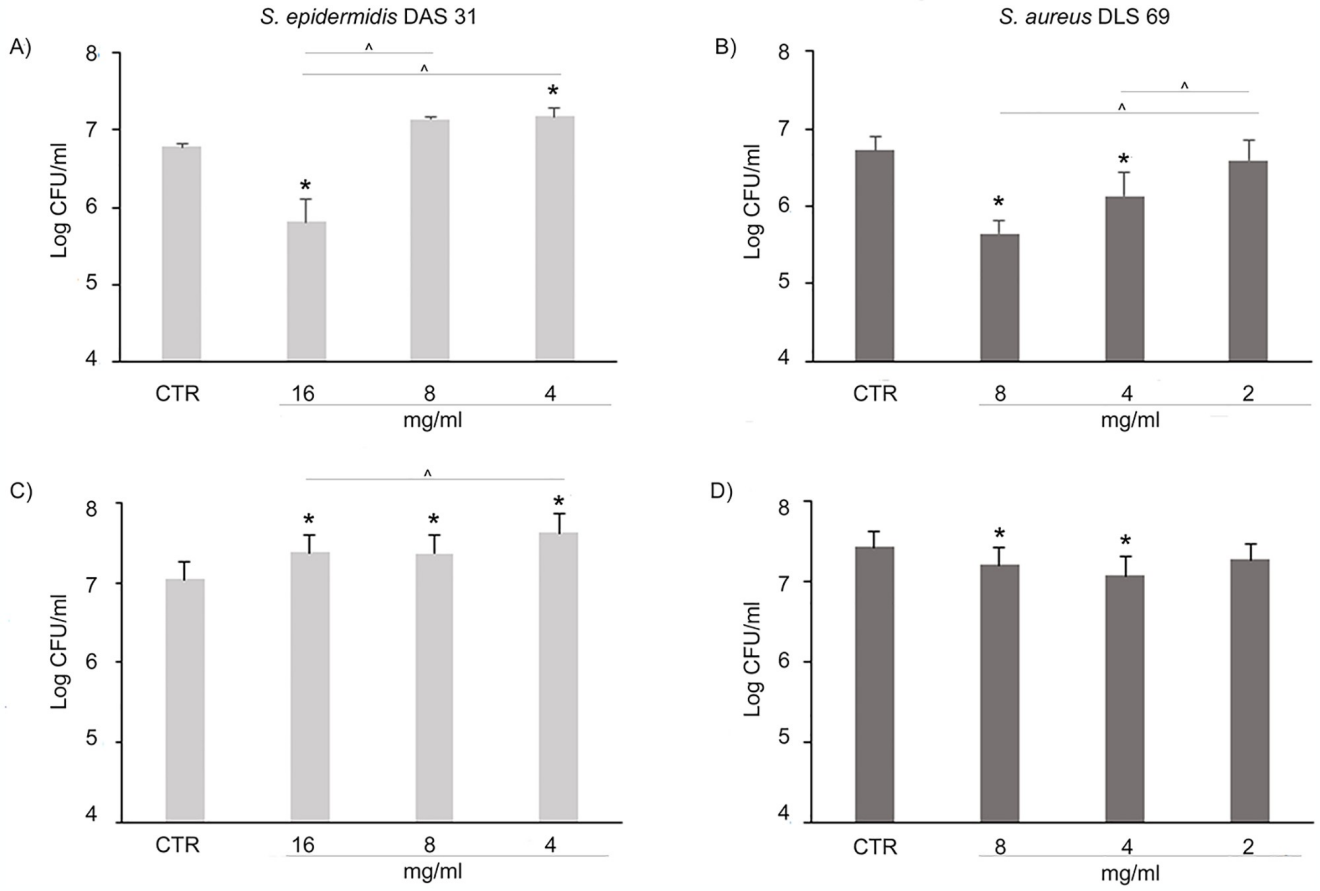
Effect of PPE in DME after 3 h and 24 h on mono-species biofilm formation. Log CFU/mL of *S*. *epidermidis* DAS 31 (**A**, **C**) and *S*. *aureus* DLS 69 (**B**, **D**) in mono-species biofilms in presence of PPE in DME at 16, 8, 4 mg/mL (1/2, 1/4 and 1/8 MIC *S*. *epidermidis* DAS 31) and 8, 4, 2 mg/mL (1/2, 1/4 and 1/8 MIC of *S*. *aureus* DLS 69) at 3 h (**A, B**) and 24 h (**C, D**). *Statistically significant in respect to the control. ^Statistically significance among the groups. (CTR: control).

Fig 3 shows the PPE in DME anti-adhesive effect against mono-species biofilm. *S*. *epidermidis* DAS 31 biofilm significantly increased in terms of CFU/mL at 1/8 MIC (4 mg/mL) after 3 h (Fig 3A) and at all tested concentrations after 24 h (Fig 3C) in respect to the control. A significant *S*. *aureus* DLS 69 CFU/mL reduction was recorded at 1/2 MIC and 1/4 MIC (8 mg/mL and 4 mg/mL respectively) after 3 h and 24 h in respect to the control (Fig 3B and 3D). At these concentrations the PPE in DME was able to affect in a species-specific way *S*. *aureus* DLS 69 promoting the *S*. *epidermidis* DAS 31 growth.

**Fig 4:**
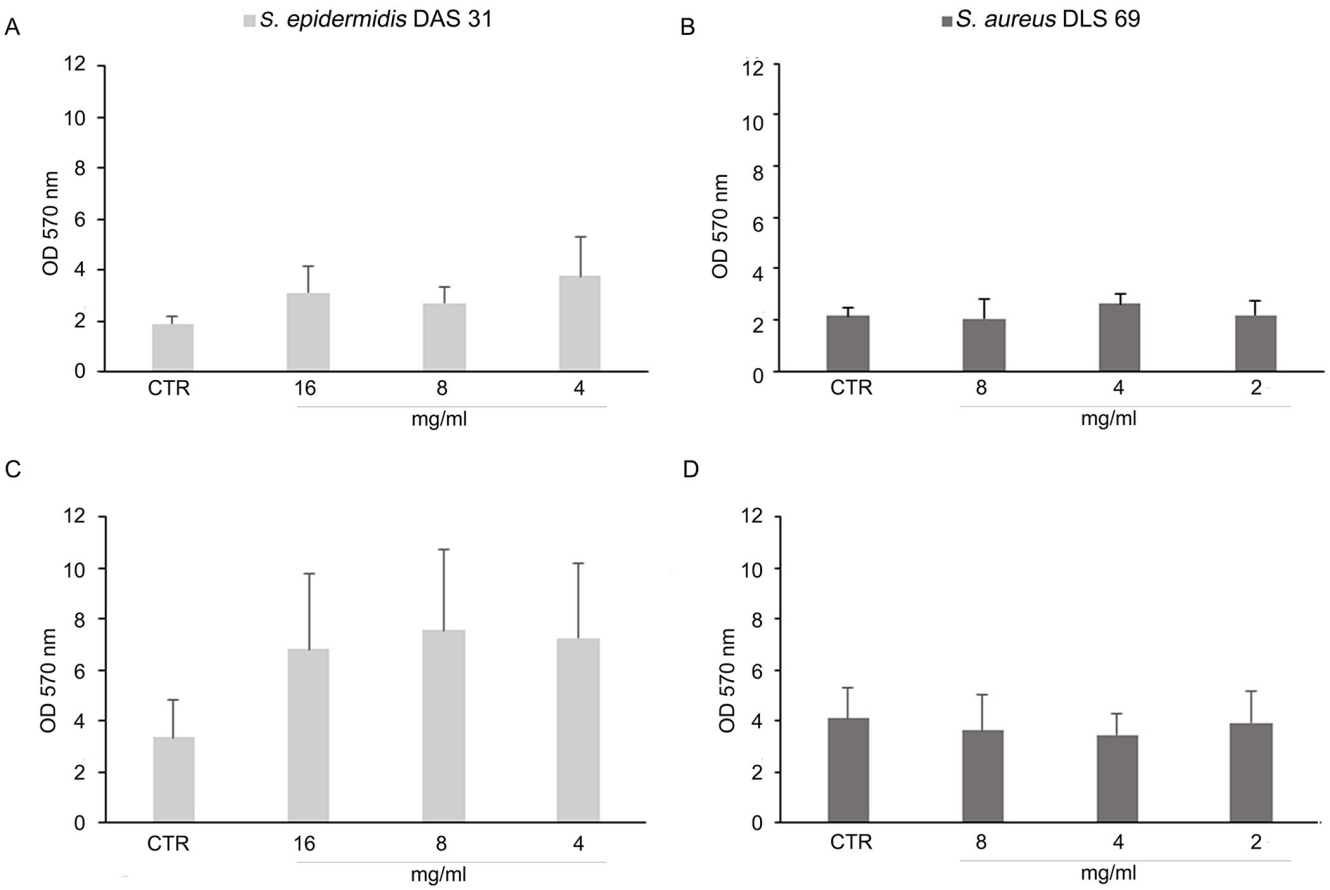
Effect of PPE in DME after 3 h and 24 h on mono-species biofilm formation. *S*. *epidermidis* DAS 31 (**A**, **C**) and *S*. *aureus* DLS 69 (**B**, **D**) biofilm biomass after treatment with 16, 8, 4 mg/mL (1/2, 1/4 and 1/8 MIC *S*. *epidermidis* DAS 31) and 8, 4, 2 mg/mL (1/2, 1/4 and 1/8 MIC of *S*. *aureus* DLS 69) of PPE in DME at 3 h (**A, B**) and 24 h (**C, D**).

As shown in Fig 4, after 24 h, PPE in DME showed a remarkable anti-adhesive effect on *S*. *aureus* DLS 69 mono-species biofilm at 8 mg/mL and 4 mg/mL with a 12.2% and 16.2% of biomass reduction, respectively, without interfering with *S*. *epidermidis* DAS 31 growth (*S*. *aureus* DLS 69 OD CTR_570_ = 4.1 ± 1.2; *S*. *aureus* DLS 69 treated with 8 mg/mL PPE in DME = 3.6 ± 1.4; *S*. *aureus* DLS 69 treated with 4 mg/mL PPE in DME = 3.4 ± 0.9).

**Fig 5:**
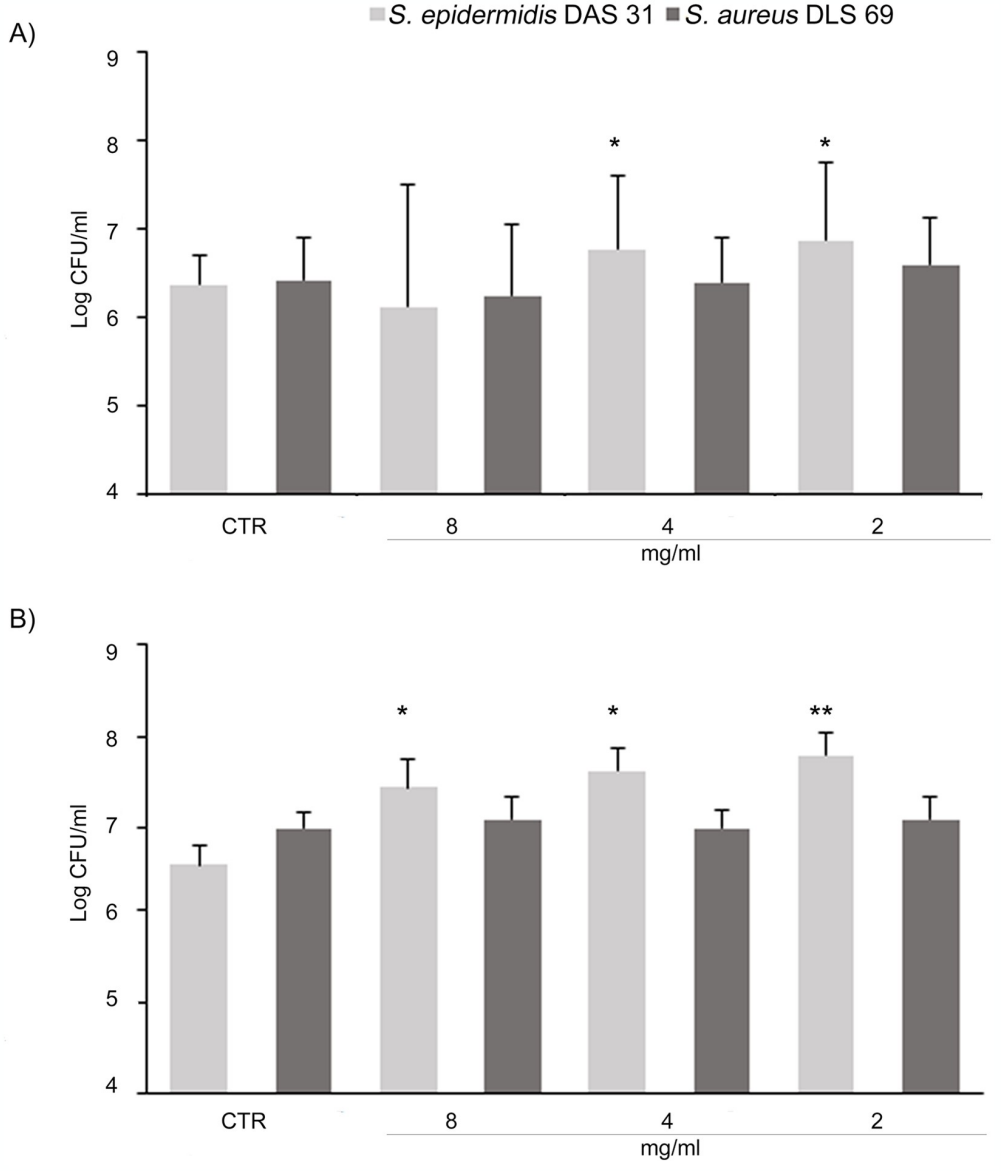
Effect of PPE in DME after 3 h and 24 h on dual-species biofilm formation. Log CFU/mL of *S*. *epidermidis* DAS 31 and *S*. *aureus* DLS 69 in dual-species biofilm, in presence of 8, 4, 2 mg/mL (1/2, 1/4 and 1/8 MIC) PPE in DME after 3 h (**A**) and 24 h (**B**). *Statistically significant in respect to the control. (CTR: control).

Fig 5 shows the Log CFU/mL of dual species biofilm after treatment with 1/2 MIC (8 mg/mL), 1/4 MIC (4 mg/mL), 1/8 MIC (2 mg/mL) of PPE in DME at 3 h and 24 h. A significant CFU/mL increase of *S*. *epidermidis* DAS 31 was recorded at 4 mg/mL and 2 mg/mL after 3 h and 24 h and at 8 mg/mL after 24 h.

### Toxicity test

**Fig 6:**
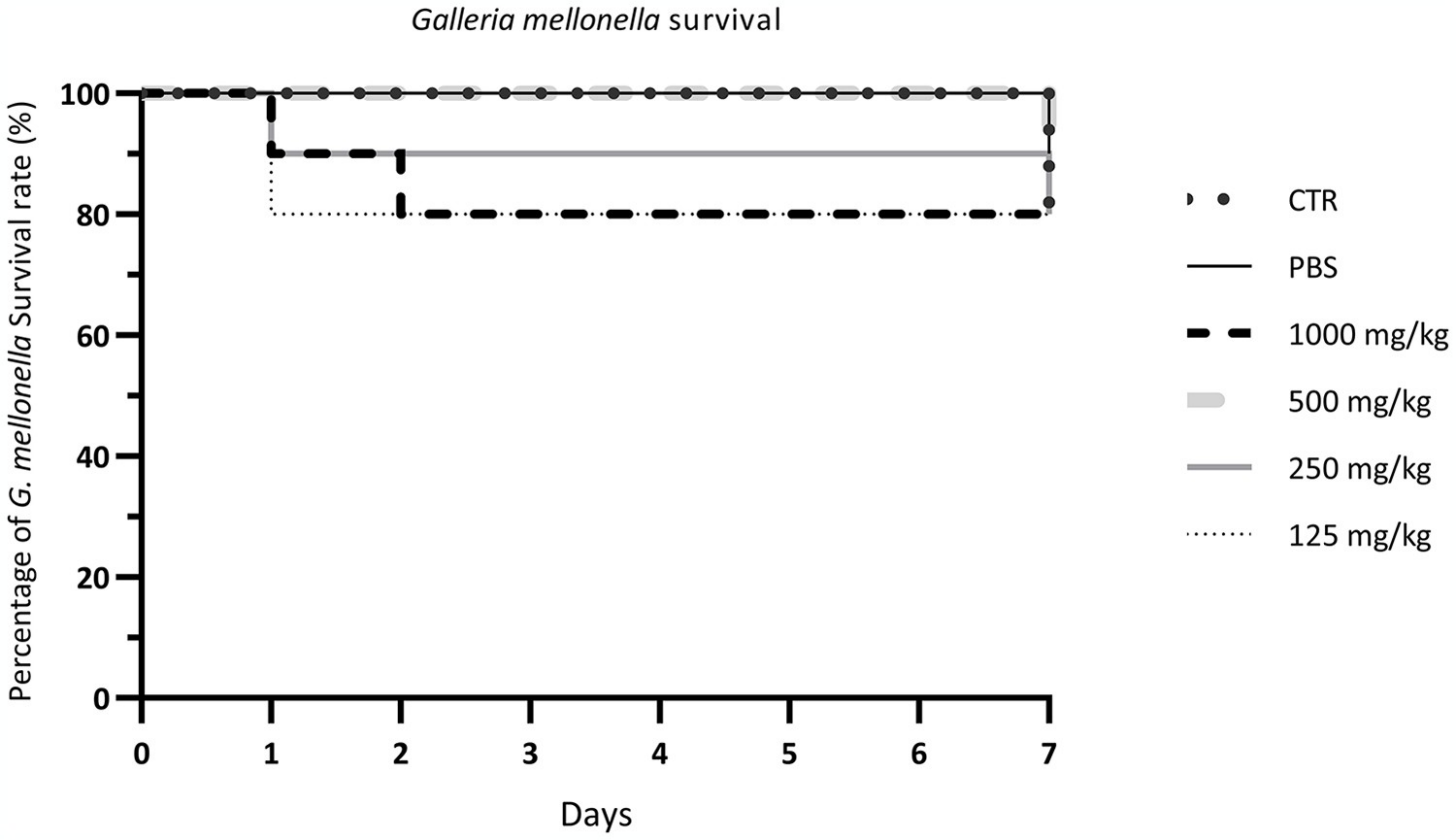
*In vivo* toxicity assay. Survival rate of *Galleria mellonella* larvae after treatment with PPE in DME, shown through Kaplan-Meier curve.

The PPE in DME toxicity was evaluated at different concentrations in *G*. *mellonella* larvae *in vivo* model at different concentrations. As shown in Fig 6, the survival rate of larvae at the highest concentration (1000 mg/kg) was 90% after 1 day and 80% after 7 days. The survival rate of larvae treated with PPE in DME was similar to the larvae treated with PBS, demonstrating the non-toxic action of PPE in DME. At lower concentrations (125 mg/mL, 250 mg/mL and 500 mg/mL), the survival rate ranged from 80 to 90%. After 7 days, the control showed a survival rate of 80%.

## Discussion

Human skin is an ecosystem that includes microorganisms living in a condition of equilibrium and boosting multiple aspects of protective functions of the skin [29]. In this study, the isolated skin strains from healthy subjects are in line with previous researches about skin microbiota in eubiosis [3, 4]. Unlike literature, no significant differences were recorded between male and female subjects [30]. This discrepancy could be attributed to the limited number of subjects, almost of the same age (25–32 years old), with an overlapping lifestyle, as university students, living in urban environments. Similarly to previous data, it was recorded a difference in terms of skin microbial composition in the subject from a different geographical region [31]. A shift in the skin microbiota balance may happen when one or more strains overwhelm the others leading to loss of skin microbial homeostasis. *Staphylococcus aureus* is the most responsible for skin dysbiosis, leading to the formation of dermatological lesions which could be difficult to treat because of both the well-known antimicrobial resistance phenomenon and the biofilm production. An interesting approach to counteract this unbalanced equilibrium is to stimulate the commensal skin bacterial growth such as CoNS, that inhibits the expression of *S*. *aureus agr* gene, quorum-sensing, virulence factors production and eventually biofilm formation [15]. *Staphylococcus epidermidis* is one of the most abundant beneficial species of healthy skin microbiota. It was demonstrated the ability of *S*. *epidermidis* to produces ceramides which are important to restore the integrity of cutaneous barrier [32]. Therefore, it is important to find active compounds with a species-specific behavior, that could represent a successful strategy in restoring skin eubiosis.

In this study, it was investigated PPE antimicrobial and anti-biofilm proprieties, highlighting its eco-sustainable value related to the reuse of a food waste.

As shown in “*The Food Loss and Waste database*” by Food and Agriculture Organization of the United Nations (FAO), a very high percentage of vegetables and fruits is thrown away [33]. The edible part of the pomegranate fruit consists of 40% aryls and 10% seeds, and so the peel (the waste) represents about 50% of the whole fruit [34]. PPE showed the ability to prevent and to treat many pathological disorders, due to the high quantity of bioactive polyphenols, mostly ellagitannins, which include a wide range of organic compounds with undoubtedly interesting activity [35, 36]. In fact, bioactive phytochemicals are present not only in the edible portion, but also in the exocarp (peel, non-edible), where they are mainly concentrated [37, 38].

Polyphenols inside peel displayed beneficial effects such as antioxidant [39], anti-inflammatory [40], anti-osteoporosis [41], antidiabetic [42], anti-atherosclerosis [43], hepatoprotective [44] and wound healing activities [45]. Moreover, they exhibited effect on lipid metabolism preventing obesity [46], and antimicrobial effect [22, 47, 48]. In this study, the PPE showed a remarkable antimicrobial action probably due to the phenolic compounds. The HPLC analysis confirmed Catechin, Quercetin, Vanillic acid and Gallic acid as main detected phytochemicals. In line with our results, Ikigai et al. showed that catechins were able to damage the *S*. *aureus* and *E*. *coli* cell membrane [49].

As reported in literature, PPE showed a significant bacterial anti-adhesive effect for the presence of quercetin that is able to interfere with quorum-sensing system. Another study showed the anti-adhesive effect of quercetin against *S*. *aureus* biofilm formation that can be attributed to the extracellular pili deletion, which are crucial to the initial attachment to surfaces and suppress the expression of genes associated with adhesion and biofilm formation (*ica*A and *ica*D) and quorum-sensing gene (*agr*A) [50, 51]. Also gallic acid interfered with *S*. *aureus* biofilm formation by downregulating the *ica*A and *ica*D genes involved in the bacterium attachment to surfaces [52] and it can reduce the extracellular polymeric substances production [53]. Our results showed the species-specific action on PPE in DME on mono-species biofilm. The extract reduced the *S*. *aureus* biofilm and increase *S*. *epidermidis* biofilm. In dual-species biofilm, although no significant reduction of *S*. *aureus* biofilm was recorded, a significant increase of *S*. *epidermidis* biofilm was detected suggesting a positive species-specific activity of this natural extract. Probably, this effect is related to the capability of pomegranate peel phenolic compounds to affect genes involved in *S*. *aureus* biofilm formation interfering with the synthesis of extracellular matrix as previously reported [17].

It is important to mention the eco-sustainable extraction method, involving in a procedure which requires low temperature without solvents dispersion. Several studies highlighted the potential of using liquefied gases (LG) as solvents for extraction, such as DME [54, 55] and n-butane [56, 57]. There are numerous advantages of their use: they require low energy expenditure, they do not alter the quality of extracts and the solvent is continuously evaporated and then recycled for subsequent extractions. For these reasons, the use of LG can be considered a green extraction modality [58].

In conclusion, PPE could be considered as a valuable non-toxic strategy to restore cutaneous microbial homeostasis acting as innovative and sustainable tool to support the skin natural resilience. These findings lead to the idea that the PPE in DME could be considered as part of topical formulations using recycled waste and green extraction techniques in compliance with the One Health approach.

## Supporting information

S1 Table Strains collected in the study with their antimicrobial profile. D: Daptomycin; G: Gentamicin; L: Linezolid; O: Oxacillin; T: Tigecycline; V: Vancomycin; Te: Teicoplanin; C: Ciprofloxacin; Cl: Clindamycin; E: Erythromycin; Le: Levofloxacin; M: Meropenem; B: Benzylpenicillin; R: Rifampicin; AF: Fusidic Acid; Ce: Celftaroline; Tet: Tetracycline; TS: Trimethoprim/Sulfamethoxazole; P: Penicillin; Net: Netilmicin; Fox: Cefoxitin.
(DOCX)

S2 Table Strains collected and used in the study with their antimicrobial profile. D: Daptomycin; G: Gentamicin; L: Linezolid; O: Oxacillin; T: Tigecycline; V: Vancomycin; Te: Teicoplanin; E: Erythromycin; Le: Levofloxacin; B: Benzylpenicillin; R: Rifampicin; AF: Fusidic Acid; Ce: Celftaroline; Tet: Tetracycline; TS: Trimethoprim/Sulfamethoxazole; P: Penicillin; Net: Netilmicin; Fox: Cefoxitin.
(DOCX)

S1 Raw data (DOCX)
