# Supplementary material for: Staphylococcus aureus/Staphylococcus epidermidis from skin microbiota are balanced by Pomegranate peel extract: An eco-sustainable approach
Source: PLoS One. 2024 Aug 1;19(8):e0308211. doi: 10.1371/journal.pone.0308211 (PMC11293756; doi:10.1371/journal.pone.0308211)
Supplement: S1 Table — D: Daptomycin; G: Gentamicin; L: Linezolid; O: Oxacillin; T: Tigecycline; V: Vancomycin; Te: Teicoplanin; C: Ciprofloxacin; Cl: Clindamycin; E: Erythromycin; Le: Levofloxacin; M: Meropenem; B: Benzylpenicillin; R: Rifampicin; AF: Fusidic Acid; Ce: Celftaroline; Tet: Tetracycline; TS: Trimethoprim/Sulfamethoxazole; P: Penicillin; Net: Netilmicin; Fox: Cefoxitin. (DOCX) [file pone.0308211.s001.docx]

**S1 Table:** Strains collected in the study with their antimicrobial profile.

| **Strains** | **Antibiotic resistance profile** | | | |
| --- | --- | --- | --- | --- |
|  | Resistent | Intermediate | Sensitive |  |
| *Staphylococcus capitis*  DLS 1 |  |  | D-G-L-O-Te-T-V |  |
| *Staphylococcus capitis* DF 2 | G |  | D-L-O-Te-T-V |  |
| *Staphylococcus capitis* RRA 3 |  |  | D-G-L-O-Te-T-V |  |
| *Staphylococcus capitis* CG 4 |  |  | D-G-L-O-Te-T-V |  |
| *Staphylococcus capitis* DMA 5 |  |  | D-G-L-O-Te-T-V |  |
| *Corynebacterium tuberculostearicum* DLS 6 | B-Cl | C | L-R-V |  |
| *Corynebacterium tuberculostearicum* DAS 7 | Cl | C | B-L-R-V |  |
| *Corynebacterium tuberculostearicum* DF 8 | B-Cl | C | L-R-V |  |
| *Corynebacterium tuberculostearicum* RRA 9 | Cl | C | B-L-R-V |  |
| *Kokuria palustris* DAS 10 | \ | \ | \ |  |
| *Kokuria palustris* RRA 11 | \ | \ | \ |  |
| *Staphylococcus saccharolyticus* RRA 12 | \ | \ | \ |  |
| *Staphylococcus saccharolyticus* DLFM 13 | \ | \ | \ |  |
| *Bacillus altitudinis* DLS 14 | E | C-Le | Cl-V-L-M |  |
| *Bacillus altitudinis* RRA 15 |  | C-Le | Cl-L-E-V-M |  |
| *Staphylococcus lugdunensis* DLS 16 |  | Le | AF-B-Cl-D-E-G-O-R-Te-Tet-T-TS-V |  |
| *Staphylococcus lugdunensis* DAS 17 |  | Le | AF-B-Cl-D-E-G-O-R-Te-Tet-T-TS-V |  |
| *Staphylococcus warneri* DAS 18 |  |  | D-G-L-O-Te-T-V |  |
| *Staphylococcus warneri* CG 19 |  |  | D-G-L-O-T-V |  |
| *Staphylococcus warneri* DMA 20 |  |  | D-G-L-O-Te-T-V |  |
| *Cutibacterium acnes* DLS 21 | \ | \ | \ |  |
| *Cutibacterium acnes* DAS 22 | \ | \ | \ |  |
| *Cutibacterium acnes* DF 23 | \ | \ | \ |  |
| *Cutibacterium acnes* DLFM 24 | \ | \ | \ |  |
| *Cutibacterium acnes* DLFM 25 | \ | \ | \ |  |
| *Cutibacterium acnes* CG 26 | \ | \ | \ |  |
| *Cutibacterium acnes* CG 27 | \ | \ | \ |  |
| *Cutibacterium acnes* DMA 28 | \ | \ | \ |  |
| *Staphylococcus epidermidis* DLS 29 |  |  | D-G-L-O-T-V |  |
| *Staphylococcus epidermidis* DLS 30 |  |  | D-G-L-O-T-V |  |
| *Staphylococcus epidermidis* DAS 31 |  |  | D-G-L-O-T-V |  |
| *Staphylococcus epidermidis* RRA 32 |  |  | D-G-L-O-T-V |  |
| *Staphylococcus epidermidis* DLFM 33 |  |  | D-G-L-O-T-V |  |
| *Staphylococcus epidermidis* DLFM 34 |  |  | D-G-L-O-T-V |  |
| *Staphylococcus epidermidis* CG 35 |  |  | D-G-L-O-T-V |  |
| *Staphylococcus epidermidis* CG 36 |  |  | D-G-L-O-T-V |  |
| *Micrococcus luteus* DLS 37 | \ | \ | \ |  |
| *Micrococcus luteus* DLS 38 | \ | \ | \ |  |
| *Micrococcus luteus* DAS 39 | \ | \ | \ |  |
| *Micrococcus luteus* DF 40 | \ | \ | \ |  |
| *Micrococcus luteus*  RRA 41 | \ | \ | \ |  |
| *Micrococcus luteus* RRA 42 | \ | \ | \ |  |
| *Micrococcus luteus* DLFM 43 | \ | \ | \ |  |
| *Micrococcus luteus* DLFM 44 | \ | \ | \ |  |
| *Micrococcus luteus* DMA 45 | \ | \ | \ |  |
| *Kokuria rhizophyla* RRA 46 | \ | \ | \ |  |
| *Kokuria rhizophyla* DLFM 47 | \ | \ | \ |  |
| *Acinetobacter lwoffi* DLS 48 | \ | \ | \ |  |
| *Acinetobacter lwoffi* DMA 49 | \ | \ | \ |  |
| *Corynebacterium afermentans* DF 50 | Cl | C | B-L-R-V |  |
| *Corynebacterium afermentans* DLFM 51 | Cl |  | B-C-L-R-V |  |
| *Bacillus cereus* RRA 52 |  | C-Le | C-E-L-M-V |  |
| *Bacillus cereus* DLFM 53 |  | C-Le | Cl-E-L-M-V |  |
| *Staphylococcus hominis spp hominis* DLS 54 |  |  | D-G-L-O-T-V |  |
| *Staphylococcus hominis spp hominis* DAS 55 |  |  | D-G-L-O-T-V |  |
| *Staphylococcus hominis spp hominis* DF 56 | O |  | D-G-L-T-V |  |
| *Staphylococcus hominis spp hominis* RRA 57 |  |  | D-G-L-O-T-V |  |
| *Staphylococcus hominis spp hominis* RRA 58 |  |  | D-G-L-O-T-V |  |
| *Staphylococcus hominis spp hominis* DLFM 59 |  |  | D-G-L-O-T-V |  |
| *Staphylococcus hominis spp hominis* CG 60 |  |  | D-G-L-O-T-V |  |
| *Roseomonas mucosa* DLS 61 | \ | \ | \ |  |
| *Moraxella osloensis* DLS 62 | \ | \ | \ |  |
| *Corynebacterium durum* DF 63 | Cl-C |  | B-R-L-V |  |
| *Staphylococcus haemolyticus* CG 64 | \ | \ | \ |  |
| *Actinomyces neuii spp neuii* DLFM 65 | \ | \ | \ |  |
| *Paracoccus yeii* DF 66 | \ | \ | \ |  |
| *Bacillus idriensis* DLFM 67 |  | C-Le | E-Cl-L-M-V |  |
| *Staphylococcus aureus* DAS 68 | Cl-E | Le | AF-B-Ce-D-G-L-O-R-Te-Tet-T-TS-V |  |
| *Staphylococcus aureus* DLS 69 | L-P-Fox | E | Le-Te-V-Tet-R-Net-G |  |
| *Staphylococcus aureus* SP 70 | E-L-P-Fox | Te-Tet | Le-V-R-Net-G |  |

*D: Daptomycin; G: Gentamicin; L: Linezolid; O: Oxacillin; T: Tigecycline; V: Vancomycin; Te: Teicoplanin; C: Ciprofloxacin; Cl: Clindamycin;  E: Erythromycin; Le: Levofloxacin; M: Meropenem; B: Benzylpenicillin; R: Rifampicin; AF: Fusidic Acid; Ce: Celftaroline; Tet: Tetracycline; TS: Trimethoprim/Sulfamethoxazole; P: Penicillin; Net: Netilmicin; Fox: Cefoxitin.*
