# Supplementary material for: Staphylococcus aureus/Staphylococcus epidermidis from skin microbiota are balanced by Pomegranate peel extract: An eco-sustainable approach
Source: PLoS One. 2024 Aug 1;19(8):e0308211. doi: 10.1371/journal.pone.0308211 (PMC11293756; doi:10.1371/journal.pone.0308211)
Supplement: S2 Table — D: Daptomycin; G: Gentamicin; L: Linezolid; O: Oxacillin; T: Tigecycline; V: Vancomycin; Te: Teicoplanin; E: Erythromycin; Le: Levofloxacin; B: Benzylpenicillin; R: Rifampicin; AF: Fusidic Acid; Ce: Celftaroline; Tet: Tetracycline; TS: Trimethoprim/Sulfamethoxazole; P: Penicillin; Net: Netilmicin; Fox: Cefoxitin. (DOCX) [file pone.0308211.s002.docx]

**S2 Table:** Strains collected and used in the study with their antimicrobial profile.

| **Strains** | | **Antibiotic resistance profile** | |
| --- | --- | --- | --- |
|  | Resistent | Intermediate | Sensitive |
| *Staphylococcus epidermidis* DLS 29 |  |  | D-G-L-O-T-V |
| *Staphylococcus epidermidis* DAS 31 |  |  | D-G-L-O-T-V |
| *Staphylococcus aureus* DAS 68 | Cl-E | Le | AF-B-Ce-D-G-L-O-R-Te-Tet-T-TS-V |
| *Staphylococcus aureus* DLS 69 | L-P-Fox | E | Le-Te-V-Tet-R-Net-G |
| *Staphylococcus aureus* SP 70 | E-L-P-Fox | Te-Tet | Le-V-R-Net-G |

*D: Daptomycin; G: Gentamicin; L: Linezolid; O: Oxacillin; T: Tigecycline; V: Vancomycin; Te: Teicoplanin; E: Erythromycin; Le: Levofloxacin; B: Benzylpenicillin; R: Rifampicin; AF: Fusidic Acid; Ce: Celftaroline; Tet: Tetracycline; TS: Trimethoprim/Sulfamethoxazole; P: Penicillin; Net: Netilmicin; Fox: Cefoxitin.*
