# Supplementary material for: Staphylococcus aureus/Staphylococcus epidermidis from skin microbiota are balanced by Pomegranate peel extract: An eco-sustainable approach
Source: PLoS One. 2024 Aug 1;19(8):e0308211. doi: 10.1371/journal.pone.0308211 (PMC11293756; doi:10.1371/journal.pone.0308211)
Supplement: S1 Raw data — (DOCX) [file pone.0308211.s003.docx]

**Raw data of Figure 3A.** Log CFU/mL of *S. epidermidis* DAS 31 after 3 h.

|  | control | 16 mg/mL | 8 mg/mL | 4 mg/m |
| --- | --- | --- | --- | --- |
| Average | 6.23 | 5.42 | 6.44 | 6.83 |
| Standard deviation | 0.94 | 0.73 | 1.17 | 0.82 |

**Raw data of Figure 3B.** Log CFU/mL of *S. aureus* DLS 29 after 3 h.

|  | control | 8 mg/mL | 4 mg/mL | 2 mg/mL |
| --- | --- | --- | --- | --- |
| Average | 6.66 | 5.59 | 6.03 | 6.35 |
| Standard deviation | 0.25 | 0.24 | 0.28 | 0.57 |

**Raw data of Figure 3C.** Log CFU/mL of *S. epidermidis* DAS 31 after 24 h.

|  | control | 16 mg/mL | 8 mg/mL | 4 mg/mL |
| --- | --- | --- | --- | --- |
| Average | 6.90 | 7.18 | 7.22 | 7.41 |
| Standard deviation | 0.44 | 0.54 | 0.44 | 0.57 |

**Raw data of Figure 3D.** Log CFU/mL of *S. aureus* DLS 29 after 24 h.

|  | control | 8 mg/mL | 4 mg/mL | 2 mg/mL |
| --- | --- | --- | --- | --- |
| Average | 7.34 | 7.13 | 6.93 | 7.20 |
| Standard deviation | 0.24 | 0.24 | 0.39 | 0.29 |

**Raw data of Figure 4A.** OD_570_ of *S. epidermidis* DAS 31 biomass after 3 h.

|  | control | 16 mg/mL | 8 mg/mL | 4 mg/mL |
| --- | --- | --- | --- | --- |
| Average | 1.90 | 3.12 | 2.73 | 3.75 |
| Standard deviation | 0.32 | 1.02 | 0.58 | 1.55 |

**Raw data of Figure 4B.** OD_570_ of *S. aureus* DLS 29 biomass after 3 h.

|  | control | 8 mg/mL | 4 mg/mL | 2 mg/mL |
| --- | --- | --- | --- | --- |
| Average | 2.16 | 2.06 | 2.62 | 2.20 |
| Standard deviation | 0.32 | 0.79 | 0.37 | 0.54 |

**Raw data of Figure 4C.** OD_570_ of *S. epidermidis* DAS 31 biomass after 24 h.

|  | control | 16 mg/mL | 8 mg/mL | 4 mg/mL |
| --- | --- | --- | --- | --- |
| Average | 3.35 | 6.82 | 7.57 | 7.27 |
| Standard deviation | 1.48 | 2.96 | 3.13 | 2.94 |

**Raw data of Figure 4D.** OD_570_ of *S. aureus* DLS 29 biomass after 24 h.

|  | control | 8 mg/mL | 4 mg/mL | 2 mg/mL |
| --- | --- | --- | --- | --- |
| Average | 4.11 | 3.61 | 3.44 | 3.94 |
| Standard deviation | 1.19 | 1.42 | 0.86 | 1.24 |

**Raw data of Figure 5A.** Log CFU/mL of mixed biofilm of *S. epidermidis* DAS 31 and *S. aureus* DLS 69 after 3 h.

|  | control | | 8 mg/mL | | 4 mg/mL | | 2 mg/mL | |
| --- | --- | --- | --- | --- | --- | --- | --- | --- |
|  | *S. epidermidis* DAS 31 | *S. aureus* DLS 69 | *S. epidermidis* DAS 31 | *S. aureus* DLS 69 | *S. epidermidis* DAS 31 | *S. aureus* DLS 69 | *S. epidermidis* DAS 31 | *S. aureus* DLS 69 |
| Average | 6.12 | 6.25 | 5.83 | 5.95 | 6.44 | 6.32 | 6.55 | 6.51 |
| Standard deviation | 0.56 | 0.44 | 0.69 | 0.58 | 0.81 | 0.27 | 0.67 | 0.28 |

**Raw data of Figure 5B.** Log CFU/mL of mixed biofilm of *S. epidermidis* DAS 31 and *S. aureus* DLS 69 after 24 h.

|  | control | | 8 mg/mL | | 4 mg/mL | | 2 mg/mL | |
| --- | --- | --- | --- | --- | --- | --- | --- | --- |
|  | *S. epidermidis* DAS 31 | *S. aureus* DLS 69 | *S. epidermidis* DAS 31 | *S. aureus* DLS 69 | *S. epidermidis* DAS 31 | *S. aureus* DLS 69 | *S. epidermidis* DAS 31 | *S. aureus* DLS 69 |
| Average | 6.50 | 6.94 | 7.16 | 6.98 | 7.47 | 6.92 | 7.58 | 6.98 |
| Standard deviation | 0.34 | 0.22 | 0.60 | 0.34 | 0.43 | 0.30 | 0.56 | 0.33 |
